# Challenging Race-Based Medicine Through Historical Education About the Social Construction of Race

**DOI:** 10.1089/heq.2023.0036

**Published:** 2023-11-30

**Authors:** Allison L. Skinner-Dorkenoo, Kasheena G. Rogbeer, Apoorva Sarmal, Cynthia Ware, Jennifer Zhu

**Affiliations:** ^1^ Department of Psychology, University of Georgia, Athans, Georgia, USA.; ^2^ Department of Psychology, University of California Los Angeles, Los Angeles, California, USA.

**Keywords:** race-based medicine, health disparities, race correction

## Abstract

**Background:**

Scientifically, there is little genetic variation among humans and race has no biological basis. However, medical school preclinical curricula tend to misrepresent race and reify biologically essentialist explanations for disease. The social construct of race is, therefore, used to inform health care providers' treatment decisions. Use of race-based medicine has been identified as a contributor to racial health disparities, spurring a growing movement to challenge race essentialism and race-based medicine. The current research tested an intervention that educates college students about the historical construction of racial categories in the United States.

**Methods:**

Participants who were randomly assigned to the intervention condition read an article highlighting the history of the sociopolitical construction of race. They were then prompted to discuss in dyads how racial categories were created and changed over history, and—in light of all this—the appropriateness of race-based medicine. Those assigned to the control condition advanced directly to the outcome measures.

**Results:**

Participants in the intervention condition reported less race essentialism, less support for race-based medicine, and greater belief that race-based medicine contributes to racial health disparities. Findings were not moderated by premed status.

**Discussion:**

Our data provide initial evidence that our interactive intervention could effectively reduce biological essentialism and support for race-based medicine in both premed and non-premed students.

**Health Equity Implications:**

This intervention has the potential to shape the way health care providers in-training understand race, their internalization of biologically essentialist explanations for disease, and willingness to adopt race-based treatment plans.

## Introduction

The concept of race emerged during the colonial era, as European slave traders were looking to justify their enslavement of African people.^1^ In the United States, White philosophers, scientists, and physicians justified the system of chattel slavery by arguing that Black people were genetically inferior, which predisposed them to enslavement.^2–4^

By the turn of the 20th century, beliefs in biologically distinct races of people were firmly entrenched in U.S. society and these distinctions were increasingly used to explain the racial disparities that resulted from generations of systemic racial oppression.^5,6^

A host of systemic factors—from environmental racism and the biological weathering that stems from repeated experiences of discrimination, to race-based medicine—contribute to the alarming racial health disparities observed today.^7,8^ Yet, essentialist explanations that tie racial health disparities to fundamental biological differences persist.^6^

Racial essentialism—the idea that racial categories are biologically distinct from one another—is so ingrained in U.S. society that attempts to dispel the myth that race is rooted in genetics are often unsuccessful.^9,10^ Formal socialization into the belief that race is genetically determined begins in primary school through science classes that fail to contextualize associations between race and disease.

For example, biology textbooks teach students to associate medical conditions such as sickle-cell anemia with Black people,^11,12^ without explaining the underlying reason for the seeming association between certain health conditions and race. In the case of sickle-cell anemia, it is linked to the historical prevalence of malaria in a region and impacts people with ancestry from a variety of geographical areas, including much of Africa, India, the Caribbean, the Middle East, and the Mediterranean.^13^

Continuous exposure to such messages, which emphasize biological distinctions between racial groups, can increase race essentialism over time.^14–17^ Thus, by the time U.S. students matriculate into medical school most of them already conceptualize race in a biological way. This conceptualization is further reinforced by the tendency for medical school curricula to emphasize race as a central factor in determining patient care, treatment, diagnosis, and medical research.^18^

Despite advanced genomic research confirming that there is little genetic variation among humans, and much more genetic variation within racial groups than there are between them,^19^ race continues to be central to U.S. medical training. Race is frequently presented as a biological risk factor for disease in preclinical lectures at U.S. medical schools^18–20^ and students are often encouraged to memorize race-disease pairings,^18,21^ which are useful diagnostic shortcuts for the U.S. Medical Licensing Examination Step 1.^22^ In fact, a substantial minority of medical students and residents report false beliefs about biological differences between racial groups—such as Black people having thicker skin than White people.^23^

Biological notions of race have also been formalized in the medical system, such that many tests and treatments are “corrected” for race. Such “corrections” are used in a variety of areas of medicine, from endocrinology and nephrology to oncology and obstetrics, which often have the effect of requiring patients of color to be sicker before they qualify for the same level of care as a White patient.^24–26^

For instance, a recent analysis of the impact of removing race corrections in the treatment of kidney disease indicated that a third of Black kidney disease patients in the United States would receive an elevated level of care if the race corrections were dropped.^27^ Similarly, Black and Hispanic women's maternal morbidity and mortality rates are elevated by race corrections in the vaginal birth after the cesarean clinical algorithm, which recommend that they opt for surgery.^28^

Given all of this, race-based medicine has been identified as a contributor to racial health disparities in the United States ^29^ and a growing minority of medical students, physicians, scholars, and researchers are calling for the medical field to revise their essentialist conceptualizations of race and abolish race corrections.^28,30–35^

Some major medical organizations, such as the American Medical Association, have responded to this scrutiny by reconsidering how medical education, research, and practice contribute to racial health disparities, and by adopting policies that aim at reducing racial essentialism in medicine.^36^

Yet, given how embedded biological notions of race are in medical diagnosis and treatment in the United States, both structural and psychological changes will be needed to eradicate race-based medicine. Since the premises of race-based medicine stand upon essentialist notions of race, educating future health care professionals about the history of the sociopolitical construction of race may be a critical step in inducing psychological change.

This theoretical perspective conceptually builds upon the epistemologies of ignorance framework,^37–40^ wherein ignorance of highly relevant sociohistorical background information is theorized to shape contemporary perceptions of societal issues. If medical students correctly understand race as a social and political category (as opposed to a biological category), encountering treatment protocols or recommendations that vary based on race should lead to skepticism.

Prior research shows that education can be a viable pathway to reduce biological essentialism.^41^ In their study, Tawa recruited participants from social media platforms to watch a 10-min video challenging the biological validity of race and emphasizing its sociopolitical construction. Participants who were exposed to this video showed reduced biological essentialism relative to the control group (who watched a video about stereotyping).

Building upon Tawa's research, we designed an interactive discussion-based intervention that employs active learning strategies to teach students about the history of the sociopolitical construction of race and the harms of race-based medicine. Active learning has been identified as the most effective approach to learning across multiple domains and disciplines,^42^ and numerous studies have shown that interactive engagement, wherein participants discuss concepts with peers (rather than just passively receiving new information), resulted in dramatically better learning outcomes.^43–45^ Beyond facilitating deep learning, active learning activities also seem to facilitate long-term changes in attitudes.^46–48^

### Current study

In the current study, we tested the potential for an active learning intervention that educated U.S. undergraduates about the sociopolitical history of the construction of race to reduce biological essentialism and decrease support for race-based medicine. Specifically, we educated U.S. undergraduates about the historical construction of racial categories in the United States and the ways in which they have changed over the course of history to meet sociopolitical goals (e.g., the “one drop rule” and the “Pocahontas Exception”).

After reading an article on the sociopolitical construction of race, participants were guided through a dyadic discussion with another participant. The semi-structured discussion encouraged participants to build personal connections with the material presented and link it to their prior knowledge—a process referred to by psychologists as cognitive elaboration and deemed important for deep learning to occur.^49,50^

Participants also read about the personal experience of a biracial teenage boy whose health was harmed by the use of race correction, given evidence that sharing subjective experiences is particularly persuasive in shaping perspectives on social issues.^51^ Our intervention approach was developed using the Interactive, Constructive, Active, and Passive framework to facilitate interactive learning among students, which has been identified by education scholars as the strategy that results in the most learning.^52^

Through our structured discussion prompts, we sought to both (1) facilitate an understanding of the sociopolitical construction of race and (2) encourage critical consideration of the suitability of using a sociopolitical distinction in treating biological conditions. We recruited U.S. college students because they have generally been exposed to essentialist notions of race through both cultural messages^53^ and formal education,^11,12^ and a substantial proportion of the students in the selected university participant pool intend to apply to medical school.

Testing our interactive educational intervention in this population allowed us to do an initial assessment of its potential effectiveness (proof of concept) before attempting a much more costly and logistically challenging intervention with medical students.

## Methods

### Participants

Undergraduate students (*N*=356) were recruited from the psychology department participant pool at a large public university in the Southeastern United States to complete this study on Zoom. Demographic details broken down by condition are provided in Table 1. Study materials and procedures were approved by the Institutional Review Board at the University of Georgia, and all participants provided informed consent before completing the study.

**Table 1.** Sample Demographics by Experimental Condition

	Control (***n***=161)	Intervention (***n***=195)
Mean age in years (SD)	18.97 (1.33)	18.73 (1.01)
Gender identity (%)		
Woman	72.67	70.62
Man	24.22	27.32
Another gender identity	3.11	2.06
Premed (%)	32.70	34.54
Racial identity (%)		
White	65.22	68.21
Black	9.94	9.74
Native American	0	0
Asian	16.77	15.38
Multiracial	6.83	4.10
Another racial group	1.24	2.56
Ethnically identifies as Hispanic (%)	6.83	6.19

SD, standard deviation.

### Procedure

We used the Zoom video conference program to conduct this study. For each session, there was one experimenter charged with providing instructions to the participants and another who managed Zoom operations (e.g., admitting participants to the call, de-identifying their screen name, placing them in breakout rooms, recording the session).

The chat was disabled to prevent participants from privately communicating with each other via text. Participants were recruited in pairs, and a modified random assignment was used to place participants in either the intervention or the control condition. Random assignment was used to ensure that individual differences among participants (e.g., in their knowledge of race and its conceptualization as biological) before the study were evenly distributed across conditions, such that any observed differences on our measures between groups could be attributed to our intervention.

**FIG. 1.**
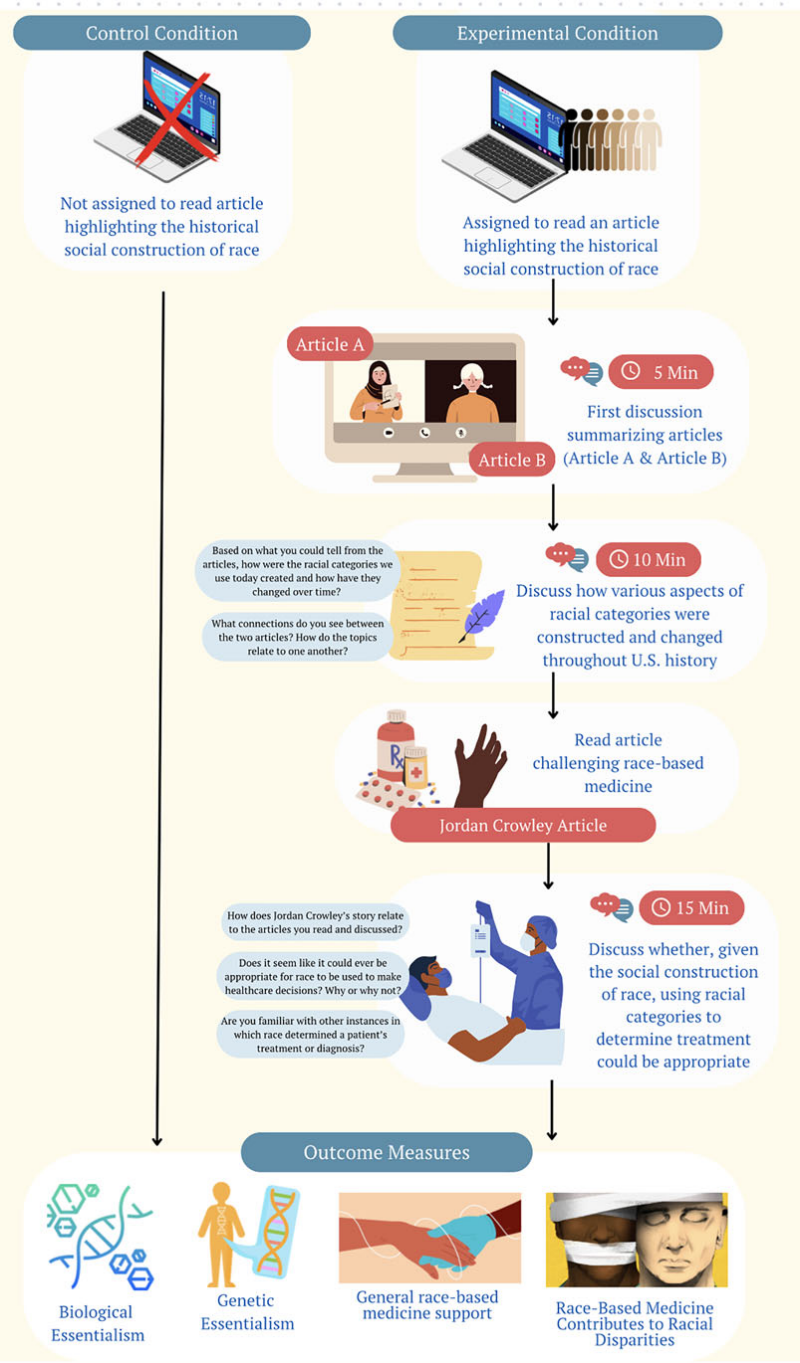
Visual representation of the study procedure.

Two participants were required to administer the experimental condition; therefore, when only a single participant was present to participate in the session, they were placed in the control condition regardless of their originally planned condition assignment. See Figure 1 for a flowchart of the procedure.

Those assigned to the control condition went directly to the outcome measures. Those who were assigned to the intervention condition each read a different article (∼500 words) highlighting the historical social construction of race (see the Open Science Framework for full articles, materials, and data) and then spent 5 min sharing the information they had read with one another.

Next, they were prompted to discuss how U.S. racial categories were created (3 min) and how categories have changed throughout history (3 min). Then, both participants read an article (∼500 words, referred to as the “Jordan Crowley article”) about a biracial teenage boy who was denied medical treatment as a result of the “race correction” that his doctors applied after deciding to categorize him as Black.

Finally, participants discussed connections between the first and second articles they read (3 min), whether race-based medicine could ever be appropriate (3 min), and other examples of race-based medicine of which they were aware (3 min).

After the discussion task, participants completed a series of measures, which were presented in random order. Essentialism measures were assessed on a 7-point Likert scale ranging from 1 (strongly agree) to 7 (strongly disagree). Biological race essentialism (*α*=0.86)^54^ included items such as, “I think the chief reason why people of a particular race are so alike in their behavior is that they possess a shared genetic inheritance.”

General race essentialism was assessed using the Race Conceptions Scale^55^ (*α*=0.84) and included items tapping into perceptions of race as biological and fixed over time and across cultures. We assessed support for race-based medicine in general (*α*=0.90), and trust in (*α*=0.81) and agreement with (*α*=0.78) specific race-based medical decisions (see Table 2).

**Table 2.** Questions About Responses to Race-Based Medicine Created for This Study

Perceptions of race-based medicine
A. Attitudes toward race-based medicine 5-point scale from “strongly agree” to “strongly disagree” 1. It is appropriate for doctors to consider the race of their patients when prescribing medical treatments. 2. Algorithms used to make health risks assessments should be adjusted based on the race of the patient. 3. It makes sense that medical devices that measure biological functioning should use different calculations for people of different races. 4. It is appropriate for medical diagnoses and treatments to vary according to the patient's race. 5. Preventive health care should be tailored to patients based on their race. B. Race-based medicine scenarios 7-point scale of *agreement* with the doctor's decision/recommendation from “strongly agree” to “strongly disagree” 7-point scale of *trust* in the doctor's decision/recommendation from “to an extremely small extent” to “to an extremely large extent” 1. Jamaal goes to the doctor to get his kidney function tested. Although the lab results indicate that Jamaal's kidney function might be deteriorating, these results did not account for Black people's greater muscle mass and bone density. After making race corrections, the doctor decides that there is nothing to worry about. 2. At her last annual medical checkup, Vedika's BMI indicated that she was at the appropriate weight for her height. However, her primary physician suggested that Vedika cuts down on sugars and fats because, as an Asian woman, her higher visceral body fat placed her at increased risk for diabetes. 3. Jazmin has been suffering from hypertension for quite some time and decided to ask her primary care physician to prescribe an ACE inhibitor medication that helps manage hypertension. However, the doctor refused to prescribe the medication on the basis that they are less effective in Black patients and may impact her cardiovascular functioning. 4. Camila had a cesarean section for her first child but wanted to deliver her second child through vaginal birth. To advise Camila on her decision, her OB-GYN used the VBAC algorithm, which takes into account Camila's Hispanic identity. Based on the VBAC indicating that Camila had lower chances of successful vaginal birth, her OB-GYN advised against vaginal birth. 5. Both Molly and Aliyah have recently been suffering from arthritis, reporting similar levels of pain. During their last doctor appointments, they were both screened for osteoporosis and received the same risk estimation. However, Molly's risk estimation was bumped up by five points to correct for her White racial identity. The new score placed Molly in the high-risk category, prompting her doctor to conduct further medical examinations and intervene early.

ACE, angiotensin-converting enzyme; BMI, body mass index; VBAC, vaginal birth after cesarean.

We also measured beliefs that race-based medicine contributes to racial health disparities on a scale of 1 (strongly agree) to 7 (strongly disagree) using the following item: “Please respond with the degree to which you personally disagree/agree that race-based medicine contributes to racial disparities in health.” Lastly, participants completed demographic measures, were debriefed about the purposes of the study, and were awarded course credit for their participation.

All measures (except for trust in race-based medicine) were reverse-scored before analysis, such that higher values indicated more essentialism, support, agreement, and trust in race-based medicine, and stronger beliefs that race-based medicine contributes to racial disparities. We also assessed cultural essentialism^54^ and racial colorblindness, but do not present results here because they are beyond the scope of this manuscript. A more detailed description of our procedure and measures can be found in the Supplementary Materials S1.

## Results

**FIG. 2.**
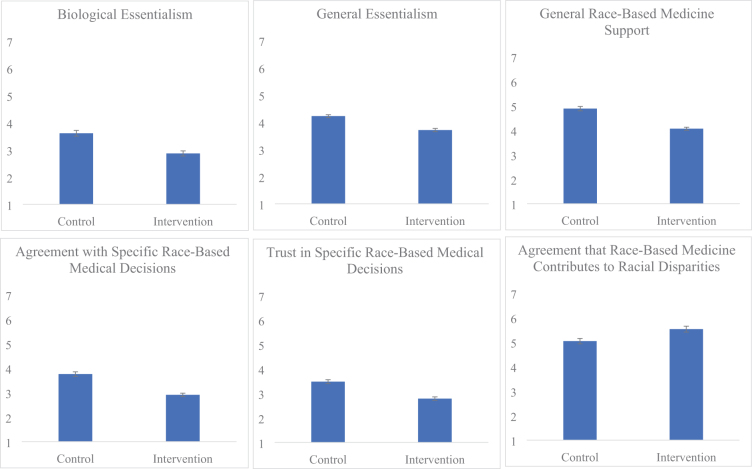
Means and standard errors by condition. Higher values indicate more essentialism, support/agreement/trust in race-based medicine, and agreement that race-based medicine contributes to racial health disparities.

To assess whether our intervention impacted participants' attitudes, we conducted a series of *t*-tests comparing means across conditions (see Fig. 2). The intervention and control condition of participants significantly differed from one another on all six of our outcome measures (see Table 3).

**Table 3.** Mean Estimates and Standard Errors for Outcome Variables in Each Condition, and *t*-Test Results Comparing Condition Means

Outcome measure	Control	Intervention	***t***-Value	***p***-Value
Biological Essentialism	3.61 (0.11)	2.87 (0.09)	5.25	<0.001
General Essentialism	4.22 (0.05)	3.71 (0.06)	6.77	<0.001
General Race-Based Medicine Support	4.88 (0.09)	4.06 (0.06)	7.52	<0.001
Agreement with Specific Race-Based Medical Decisions	3.76 (0.09)	2.91 (0.07)	7.31	<0.001
Trust in Specific Race-Based Medical Decisions	3.48 (0.08)	2.79 (0.07)	6.45	<0.001
Race-Based Medicine Contributes to Racial Health Disparities	5.04 (0.11)	5.53 (0.12)	2.92	0.004

We also conducted analysis of variances (ANOVAs) to examine whether premed status predicted our outcomes or moderated the effects of the intervention. Premed students (*M*=3.56, *SE*=0.10) were more likely to agree with the physicians' race-based medical decision than non-premed students (*M*=3.21, *SE*=0.07), *F*(1, 349)=7.79, *p*=0.006.

Relative to non-premed students (*M*=3.02, *SE*=0.07), premed students (*M*=3.37, *SE*=0.09) were also more likely to trust the physicians in the race-based medicine scenarios, *F*(1, 349)=10.08, *p*=0.002. Premed status did not significantly predict responses on any of the other outcome measures (*p*s>0.05), and none of the effects of condition were moderated by premed status (*p*s>0.10).

## Discussion

Structural changes to medical education and training are undoubtedly necessary to eradicate race-based medicine, but psychological changes are also needed to eradicate the attitudes that perpetuate race-based medicine. In the current study, we tested an intervention designed to educate students about the sociopolitical construction of race, to reduce racial essentialism, and to decrease support for race-based medicine.

Participants exposed to the intervention showed reduced general and biological race essentialism and expressed less support for race-based medical practices and treatment decisions. In addition, they reported greater agreement that race-based medicine contributes to racial health disparities.

As such, our findings serve as preliminary evidence that relatively brief interactive educational sessions of this kind could be a viable pathway to curb endorsement of race-based medical practices. Although additional research is needed, these data make us optimistic that an intervention of this kind could potentially be effective in reducing essentialist notions of race and decreasing support for race-based medical practices among future medical practitioners.

To this end, we tested whether our intervention would be as effective for college students intending to become physicians (pre-med), finding that pre-med status did not moderate the effect of our intervention on any of the outcomes measures. Nonetheless, follow-up studies will be critical to assess whether an intervention of this kind can stand up to the biological conceptualizations of race that are repeated throughout U.S. medical education.^18–22^

Because many medical students are likely missing highly relevant information about the historical and structural forces that created the concept of race, receiving and actively processing that information could alter the lens through which they conceptualize race, potentially facilitating long-term attitude change.

In recent years, student-led efforts to tackle racial essentialization in medical school curricula have led to the implementation of electives, journal clubs, and speakers' series that tackle the connection between the sociopolitical construction of race and medicine.^56^

However, these types of efforts are likely to attract individuals who are already concerned by the use of race as a proxy for upstream determinants of health and therefore limited in their reach.^30^ Our intervention—which takes only 30 min and could be implemented within a single class period—could be integrated into courses that all medical students are required to complete.

If established as part of the general medical school curricula, our intervention may have the potential to reduce essentialist perceptions of race among future physicians. By changing the narrative around race in medical schools, our intervention could also tackle commonly held misperceptions that race-based medicine provides better and more accurate care to people of color.^35^

Although the findings of this study are promising, it is important to acknowledge its limitations. Random assignment was used to minimize pre-existing differences between groups, but we did not assess the participant's pre-intervention attitudes; therefore, our ability to confirm that individual differences were evenly distributed is limited.

Since this was not a longitudinal study, we are also unable to draw conclusions about the durability of the attitude changes observed in our study. Additional research is needed to assess whether these attitude changes will persist over time, given the proliferation of cultural and societal messages that reinforce biological conceptions of race.^14–17^

## Health Equity Implications

U.S. medical education often portrays race as a biological factor that determines health risk and explains health disparities between racial groups.^56^ In doing so, medical schools cultivate an essentialist notion of race among their students, which increases their likelihood of using race to make medical diagnoses and treatment recommendations.^12^ For example, doctors who believe Black people have thicker skin and less sensitive nerve endings are less likely to prescribe pain medication to Black patients.^35^

This tendency to take race into account when making decisions about patient care can contribute to racial health disparities.^12^ Race-disease associations also impact research funding allocations, such that research on White-associated diseases (e.g., cystic fibrosis) can receive 3.5 times as much funding as research on Black-associated diseases (e.g., sickle cell anemia).^57^

Given the numerous negative downstream consequences of racial essentialism in medical education, scholars have argued that the medical school curricula should be revamped.^18,35^ Our intervention may be one such avenue for changing how medical education is structured, as it shows the potential to reduce racial essentialism and support for race-based medical practices among the next generation of medical practitioners.

This intervention has the potential to shape the way health care providers in training understand the concept of race, and the extent to which they internalize racially essentialist messages that they are likely to be exposed to during their medical training. As such, this approach has promising potential to help address one known contributor to racial health disparities—the utilization of race-based medicine.

By including critical historical education about the sociopolitical construction of race in medical school curricula, interventions of this kind have the potential to help reduce persistent racial health disparities that impact the lives of millions of people of color in the United States, creating a more equitable health care system.

## Supplementary Material

Supplemental data

## Abbreviations Used

ACE: angiotensin-converting enzyme
BMI: body mass index
SD: standard deviation
VBAC: vaginal birth after cesarean
