# Supplementary material for: Challenging Race-Based Medicine Through Historical Education About the Social Construction of Race
Source: Health Equity. 2023 Nov 30;7(1):764–72. doi: 10.1089/heq.2023.0036 (PMC10698761; doi:10.1089/heq.2023.0036)
Supplement: Supplemental data [file Suppl_DataS1.docx]

**SUPPLEMENTAL MATERIALS**

**Challenging Race-Based Medicine Through Historical Education About the Social Construction of Race**

**Procedure**

We used the Zoom video conference program to conduct this study. For each session, there was one experimenter charged with providing instructions to the participants and one Zoom technician who managed Zoom operations (such as admitting participants to the call, de-identifying their screen name, placing them in breakout rooms, recording the session, etc.) The chat was disabled to prevent participants from communicating with each other than with the Zoom tech and experimenter. Participants completed this study in a dyad and were randomly assigned to either the control or intervention condition. Studies where only one participant was present, either because their partner was a no-show or no one was signed up for the same session, were reassigned to the control condition regardless of their originally planned condition assignment. Participants in the control condition proceeded directly to the outcome measures for completion.

Participants assigned to the experimental condition were placed into separate breakout rooms and provided with one of two different articles (one participant would be assigned to article A and the other to article B) designed to provide background information on the history of race, both articles can be found on [Open Science Framework](https://osf.io/fy9xk). After reading their assigned articles, participants were brought into the main Zoom conference room and instructed to keep their microphones and videos on at all times. The experimenter verbally confirmed that the participants had each other's videos pinned to ensure that they could clearly see the other participant throughout the discussion. Then, participants were given a maximum of five minutes to summarize their respective articles (Articles A and B). Following the article summary, the experimenter provided two discussion questions at three-minute intervals designed to stimulate discussion about the sociopolitical construction of race throughout history.

1. “What connections do you see between the two articles? How do the topics relate to one another?”
2. “Based on what you could tell from the articles, how were the racial categories we use today created and how have they changed over time?”

After this brief discussion, participants moved on to read an article (referred to as the “Jordan Crowley article”) about a biracial teenage boy who was denied medical treatment as a result of the “race correction” that his doctors applied after categorizing him as Black. Once both participants had privately messaged the zoom technician to confirm that they were done reading, they were provided with three discussion questions (3 minutes each), designed to probe whether using racial categories to determine medical treatment is appropriate given the sociopolitical construction of race.

1. “How does Jordan Crowley’s story relate to the articles you read and discussed?”
2. “Does it seem like it could ever be appropriate for race to be used to make healthcare decisions? Why or why not?”
3. “Are you familiar with other instances in which race determined a patient’s treatment or diagnosis?”

When the discussion session was complete, participants returned to their breakout rooms to complete the outcome measures on Qualtrics (see [Open Science Framework](https://osf.io/fy9xk) for complete measures).
